# A Novel Energy-Efficient Reservation System for Edge Computing in 6G Vehicular Ad Hoc Network

**DOI:** 10.3390/s23135817

**Published:** 2023-06-22

**Authors:** Farhan Javed, Zuhaib Ashfaq Khan, Shahzad Rizwan, Sonia Shahzadi, Nauman Riaz Chaudhry, Muddesar Iqbal

**Affiliations:** 1 Department of Electrical and Computer Engineering, COMSATS University Islamabad, Attock Campus, Attock 43600, Pakistan; farhan44933@gmail.com; 2 School of Architecture, Technology, and Engineering (ATE), University of Brighton, Brighton BN2 4AT, UK; 3 Department of Computer Science, COMSATS University Islamabad, Attock Campus, Attock 43600, Pakistan; shehzad.rizwan@cuiatk.edu.pk; 4 Department of Computer Science, University of Gujrat, Gujrat 50700, Pakistan; 18036119-002@uog.edu.pk; 5 Renewable Energy Laboratory, Communications and Networks Engineering Department, College of Engineering, Prince Sultan University, Riyadh 11586, Saudi Arabia

**Keywords:** edge computing, energy-efficient system, roadside unit, vehicular ad hoc network, vehicle-to-infrastructure, vehicle-to-vehicle, sixth-generation wireless communication

## Abstract

The roadside unit (RSU) is one of the fundamental components in a vehicular ad hoc network (VANET), where a vehicle communicates in infrastructure mode. The RSU has multiple functions, including the sharing of emergency messages and the updating of vehicles about the traffic situation. Deploying and managing a static RSU (sRSU) requires considerable capital and operating expenditures (CAPEX and OPEX), leading to RSUs that are sparsely distributed, continuous handovers amongst RSUs, and, more importantly, frequent RSU interruptions. At present, researchers remain focused on multiple parameters in the sRSU to improve the vehicle-to-infrastructure (V2I) communication; however, in this research, the mobile RSU (mRSU), an emerging concept for sixth-generation (6G) edge computing vehicular ad hoc networks (VANETs), is proposed to improve the connectivity and efficiency of communication among V2I. In addition to this, the mRSU can serve as a computing resource for edge computing applications. This paper proposes a novel energy-efficient reservation technique for edge computing in 6G VANETs that provides an energy-efficient, reservation-based, cost-effective solution by introducing the concept of the mRSU. The simulation outcomes demonstrate that the mRSU exhibits superior performance compared to the sRSU in multiple aspects. The mRSU surpasses the sRSU with a packet delivery ratio improvement of 7.7%, a throughput increase of 5.1%, a reduction in end-to-end delay by 4.4%, and a decrease in hop count by 8.7%. The results are generated across diverse propagation models, employing realistic urban scenarios with varying packet sizes and numbers of vehicles. However, it is important to note that the enhanced performance parameters and improved connectivity with more nodes lead to a significant increase in energy consumption by 2%.

## 1. Introduction

VANETs are wireless ad hoc networks that allow vehicles to interact with one another, with infrastructures, and with other highway users. VANETs have the capacity to transform the field of transportation by serving as an operating system to ensure safeguarding, improve the efficiency of traffic, and offer entertainment applications. The VANET is a well-known vehicle network with capabilities including self-configuration and self-organization. It can range from a highly dense to a very sparse topology. Along with cases of dense network topologies, the presence of vehicles on the route makes it easy to offer end-to-end multi-hop connectivity among the nodes. VANETs, however, have several limitations, including high mobility, frequent topology changes, and a restricted transmission reach. The introduction of 6G technology provides an opportunity to overcome a few of these issues and increase the VANET’s efficiency. The term “6G” refers to the sixth generation of technology for wireless communication, which is projected to deliver even faster data speeds, fewer delays, and more security than the existing 5G technology. The 6G technology is still in its early stages, with many of its characteristics and applications still being researched and developed. Despite this, a few of the most important characteristics anticipated to be incorporated in 6G include Terahertz (THz) frequencies to operate at higher frequencies than the current 5G technology [1,2], with frequencies ranging from 300 GHz to 3 THz. These high-frequency bands offer a higher bandwidth and lower latency but require new antenna designs and signal processing techniques. In addition, 6G is expected to leverage the power of AI [3] to optimize network performance, improve energy efficiency, and enable new applications, such as autonomous driving and smart cities. Moreover, the Massive MIMO [4] is anticipated to be used in 6G to increase the number of antennas in the base station and enable better beamforming and interference management. Holographic beamforming is among the techniques with the potential to enable precise directional communication and improve energy efficiency. The RSU is one of the fundamental components in VANETs, as it is the first point at which the vehicle communicates in the infrastructure. The state-of-the-art swarm of UAVs for network management in 6G, which is anticipated to offer gigantic connection, all-around coverage, ultra-reliability, and intelligence has been reviewed in this article [5].

Numerous challenges can impact the operational efficiency of VANETs, such as high mobility, frequent topology changes, and a restricted communication range. The 6G technology, with data speeds of up to 1 Tbps, has the ability to overcome some of the aforementioned obstacles and enhance the performance of VANETs in a variety of approaches. The increased data rate has the potential to support novel applications including real-time video streaming and high-definition mapping. Similarly, 6G connectivity is predicted to provide latency that is as low as 1 millisecond; 6G is also expected to provide increased dependability through greater redundancy and enhanced error correction algorithms. Even in high-mobility circumstances, enhanced dependability may ensure that communications are transmitted accurately and on time. This will also boost energy efficiency by utilizing improved power management techniques and innovative antenna layouts. This increased energy efficiency may improve the car battery life and minimize the requirement for battery replacements.

**Figure 1:**
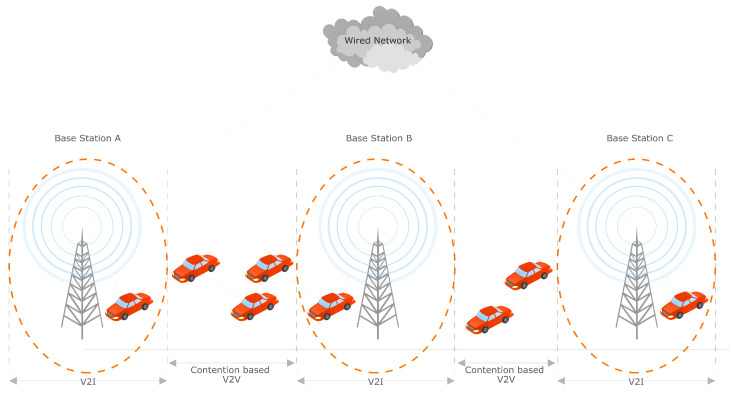
V2V and V2I communication.

The combination of VANETs and delay-tolerant networks (DTNs) has resulted in the concept of vehicular delay-tolerant networks (VDTNs) [6], which is based on the stored-carry-and-forward mechanism of DTNs and applied to the vehicular context. In order to sustain the increasing variety of intelligent transportation systems (ITS) as well as smart city applications, the VDTNs also integrate communications including vehicle-to-vehicle (V2V), vehicle-to-roadside-unit (V2R), and vehicle-to-infrastructure (V2I), as shown in Figure 1. The RSU has many functions, including the sharing of emergency messages and updating vehicles about the traffic situation.

Cars travel at fast speeds, the network topology is dynamic, and vehicles must be separated frequently. While 6G technology has the potential to increase VANETs’ effectiveness, various difficulties must be overcome before it can be extensively used. High-frequency operation is one of the main issues since 6G is planned to run at greater frequencies than the existing 5G technology, which might result in significant attenuation and signal loss. This can make maintaining consistent communication over a greater distance problematic, especially in metropolitan settings with high-rise buildings and additional obstructions. The adoption of sophisticated signal processing techniques such as holographic beamforming and large MIMO, which may boost system complexity, is also an issue. This complexity might make optimizing the system and ensuring reliable communication challenging [7]. Because 6G technology is currently in the early phase of advancement, no standardization initiatives are presently underway. This absence of standardization may contribute to market fragmentation and make ensuring compatibility across different devices and systems challenging. To guarantee that communications are safe and shielded from assault, 6G must address security issues such as authentication, encryption, and privacy.

**Figure 2:**
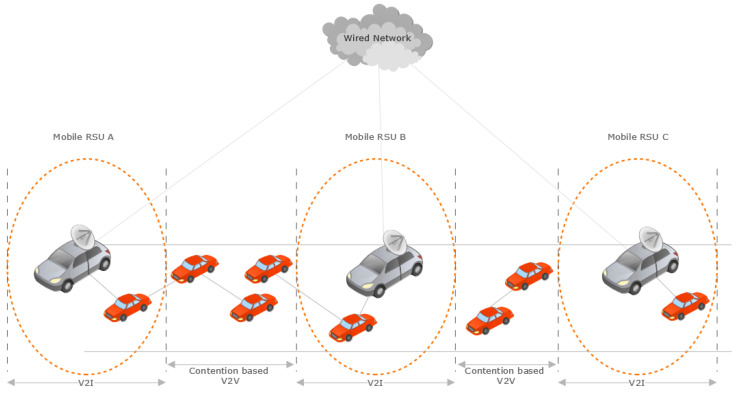
Mobile RSU.

At present, researchers remain focused on the multiple parameters to be improved in the static RSU (sRSU) to improve both V2V as well as V2I communication; however, in this paper, the research will focus on the mobile RSU (mRSU) [1], as shown in Figure 2.

The mRSU is an emerging concept that has been highlighted in some papers during the last few years. This research presents the concept of the installation of RSUs in traffic police vehicles and public transport vehicles that remain in motion during patrolling; it helps to reduce the likelihood of packet loss during communication. As police vehicles remain in motion during patrolling, they cover the same group of vehicles, and the likelihood of packet loss is reduced because of a reduction in connection breakage and the re-establishment of communication among vehicles.

Considering the multiple obstacles, 6G technology offers several opportunities in VANETs, notably the deployment of new, safety-essential features such as accident avoidance and emergency braking. Because of the low latency and great reliability of 6G technology, these features can be run in real time and offer correct knowledge to drivers. The technology will also allow for the development of novel traffic management strategies such as dynamic route optimization, traffic flow forecasting, and the real-time management of congestion. These strategies can assist in alleviating traffic congestion and enhance the overall transportation system’s performance. High-definition maps, the real-time broadcasting of videos, and interactive entertainment experiences may all be enabled with 6G [8]. The aforementioned applications have the potential to improve the experience of driving, while also providing additional revenue opportunities for vendors of services. One of the most important prospects is that 6G technology, by offering high-bandwidth and low-latency transmission between vehicles and buildings, might allow new autonomous driving technologies. Vehicles may run more effectively and safely as a result, reducing the possibility of errors and enhancing the whole system of transportation. However, by resolving the problems and capitalizing on the advantages, 6G technology has the ability to exploit the full potential of VANETs and revolutionize the transportation sector.

In the context of VANETs, edge computing [7] can be used to address the challenges associated with data processing, communication, and storage in a highly dynamic and resource-constrained environment. This research will discuss the potential benefits of edge computing in VANETs, the challenges associated with implementing edge computing in VANETs, and the current research trends in this area. One of the main advantages of edge computing is that it can significantly reduce the latency in data processing and communication. By processing data closer to the source, edge computing can eliminate the need to transmit data to a centralized server for processing, which can result in significant delays. In the context of VANETs, reduced latency is particularly important for applications that require real-time processing, such as collision avoidance and emergency response systems.

The work is organized as follows. Section 2 discusses the past research work on network simulation in the context of a vehicular environment. Section 3 delves into the proposed research methodology, both experimental and theoretical, while Section 4 discusses the results. Finally, Section 5 contains the conclusions.

## 2. Related Works

Jeongyoon et al. [9] provide two models, FMNMS1 and FMNMS2, that generate network-based judgements by taking into account characteristics including the speed of the vehicle, tyre sensitiveness, data size, geographical position, vehicle surroundings, temperature, level of noise, heart rate with ECG, breathing rate, EEG, and EOG. Meanwhile, Suresh Kumar et al. [2] recommends a framework for the management of network resources, as well as for monitoring drivers in a VANET system. The simulation model incorporates the 6G technology’s parameters and edge, fog, and cloud computing features to create a realistic and accurate representation of the system. The proposed system can handle high volumes of data with low latency and high throughput, making it suitable for real-time communication and data processing in VANETs. The concept entails the use of fuzzy logic and is regulated using edge/fog/cloud computing to enable optimal network resource management and increase road safety. The study uses a Raspberry Pi and deep learning techniques to forecast the driver’s state by collecting information from several biosensors. The system activates the actuator based on the outcome. In addition, the study features integrated edge/fog/cloud computing to solve scalability, security, seamless connection, and dependability concerns. The system presented in [3] can anticipate the relative speed, sensitivity, and traffic rate of the vehicle, as well as the driver’s state, such as sleepiness or exhaustion. Based on these data, the suggested system can park the car, notify the driver, reduce the vehicle’s speed, or shut down the engine in a critical situation. The proposed fuzzy-based VANET system intends to create a safe and user-friendly transportation infrastructure capable of reducing serious accidents and improving personal transportation security.

Wali Ullah et al. [4] present a new strategy for data sharing in 6G-VANET that utilizes blockchain and edge intelligence to improve privacy protection. The proposed approach comprises three primary elements: a data owner, a data requester, and edge nodes. The data owner encrypts data and shares them on the blockchain, while the data requester uses edge intelligence to obtain and decrypt the data. The edge nodes act as intermediaries to facilitate data sharing and enhance privacy protection by keeping the data owner’s and requester’s identities anonymous. The authors assess the proposed approach’s performance in terms of the data retrieval time, communication overhead, and privacy protection. The findings in [7] indicate that the proposed strategy outperforms current approaches in terms of data retrieval times and communication overhead, while providing robust privacy protection. Throughout the data sharing process, the identities of the data owner and requester are kept anonymous. Overall, this research introduces a promising solution for data sharing in 6G-VANET that leverages blockchain and edge intelligence to improve privacy protection while maintaining efficient data retrieval.

According to the study in [8], the current access control and security mechanisms for vehicular communication systems are insufficient in tackling the distinct issues that arise with 6G networks, such as higher data rates, reduced latency, and more extensive connectivity. The authors suggest a more elaborate approach that includes three main elements: authentication, authorization, and encryption. Authentication is responsible for verifying the user or device’s identity before granting access to the network, while authorization regulates the level of access granted to each user or device based on their privileges or roles. Encryption guarantees the confidentiality and security of all communication between users or devices. Furthermore, the proposed strategy in [10] introduces a trust management system that examines the reliability of each user or device based on their conduct and interactions with the network, thereby preventing malicious attacks or unauthorized access. The authors test their approach via simulations and experiments, concluding that it improves security and performance in vehicular communication systems within 6G networks compared to existing methods. The study presents a new access control and security approach for vehicular communication systems, addressing the specific challenges of 6G networks and offering a secure and efficient communication setting for intelligent vehicular transport in the future. An effective and reliable autonomous vehicle routing system for 6G networks with computational intelligence has been proposed in this article [11]. The protocol establishes a routing method that enhances energy optimization amongst IoT-based vehicles and ensures the ideal solution under challenging conditions [11].

The authentication scheme proposed in [12] is based on the combination of elliptic curve cryptography (ECC) and symmetric key encryption. The scheme uses both public key cryptography and symmetric key cryptography to authenticate messages transmitted between vehicles in a VANET. The scheme also employs a secure key management mechanism to generate and distribute keys among vehicles. It involves two models, a secure key management model and a message authentication model. The secure key management model [13] is responsible for generating and distributing the keys required for the message authentication model. On the other hand, the message authentication model is responsible for verifying the authenticity of the messages transmitted between vehicles. The proposed authentication scheme in [14] was tested using a simulator and the simulation results showed that the proposed scheme performed better than other authentication schemes concerning security, computational overhead, and communication overhead. The proposed scheme achieved a high level of security with a low computational overhead and low communication overhead, making it a suitable option for 6G-enabled VANETs. The research study in [15] includes several algorithms, including a key generation algorithm that is used to create the public and private keys necessary for the authentication process. The key distribution algorithm is used to securely distribute the keys among the vehicles in the VANET. The message authentication algorithm [16] is applied to authenticate the messages transmitted between the vehicles in the VANET. The key update algorithm is introduced to periodically update the keys to ensure the security of the VANET. The scheme is based on well-established cryptography techniques and employs a secure key management mechanism to guarantee the confidentiality and integrity of the messages exchanged between vehicles. The experimental results demonstrate that the proposed scheme outperforms other authentication schemes regarding security, computational overhead, and communication overhead. The research in [17] proposes an intelligent trust-based e-learning-based intrusion detection system (IDS) for vehicular ad hoc networks (VANETs) in 6G. The proposed IDS system is based on a hybrid model of machine learning and deep learning algorithms. The proposed IDS system comprises three models: a trust model, a feature extraction model, and a classification model. The trust model evaluates the trustworthiness of the vehicles in the network based on their behavior. The feature extraction model extracts features from the network data, and the classification model classifies the extracted features as normal or anomalous. The proposed system outperforms existing IDS systems [18] in terms of detection accuracy and the false alarm rate. Specifically, he proposed system achieves a high level of accuracy with a low false alarm rate, making it a suitable option for 6G-enabled VANETs. The proposed intelligent trust-based e-learning-based IDS system in [19] is also a promising solution for intrusion detection in 6G-enabled VANETs. The system is based on a hybrid model of machine learning and deep learning algorithms and comprises several algorithms for trust evaluation, feature extraction, and classification. The experimental results demonstrate that the proposed system outperforms existing IDS systems in terms of detection accuracy and the false alarm rate.

The 5G-NR, vehicle-to-everything (V2X) [20] is a technology that provides improved performance and advanced services by investing in more spectral and hardware resources. With the growth of autonomous vehicles due to urbanization, improved living standards, and technological advancements, there is an increased demand for communication devices and digital applications to enable intelligent autonomous vehicles. The emergence of many new services, such as 3D displays, holographic control display systems, and improved in-car infotainment, will bring communication challenges to the V2X network. This will push the capacity limits of existing wireless networks, posing new scientific and technical challenges for vehicular networks. Legacy V2X communication systems [21] can only provide limited intelligence; therefore, there is a need for a significant shift away from traditional communication networks to more versatile and diversified network approaches. The proposed 6G wireless communication network will combine terrestrial and non-terrestrial communication networks to enable intelligent and ubiquitous V2X systems with enhanced reliability, security, and high data rates. New techniques are needed to enable adaptive learning and intelligent decision-making in future V2X networks; 6G is expected to work with machine learning to bring enhanced context awareness, self-aggregation, adaptive coordination, and self-configuration, among other features. The concept of intelligent reflective surfaces (IRSs) [22] refers to a disruptive communication technique that has recently gained attention. IRSs are programmable meta-surfaces consisting of numerous passive antenna elements that can control the phase, amplitude, frequency, and polarization of wireless signals, improving the propagation conditions and enabling higher rates, wider coverage, and uninterrupted connectivity. In this paper, a multi-IRS and multi- unmanned aerial vehicle (UAV)-assisted MEC system for 5G/6G networks are discussed where several UAVs support numerous IRSs to serve a high number of User Equipment’s (UEs) [23]. In scenarios where V2X communications operate at the millimeter-wave or THz bands or under unfavorable propagation conditions, the use of IRSs can enhance the vehicular channel conditions and expand the transmission coverage. IRSs can also be used to mitigate the negative effects of the Doppler effect [24] and multi-path fading, making them an appealing research direction for 6G-V2X. A use case scenario for IRSs is their installation on buildings around out-of-coverage traffic intersections, enhancing the communication coverage for transmitting vehicles in the perpendicular streets by fine-tuning the reflecting elements of IRSs. To efficiently integrate them with 6G-V2X, some fundamental challenges, such as reflection optimization, the optimal placement of IRSs, channel estimation in a highly dynamic vehicular environment, and adaptation to different spectral ranges, must be overcome.

Tactile communication [25] is a new technology that allows the real-time transmission of touch, motion, vibration, and other sensory information. This technology is expected to revolutionize communications in 6G-V2X by providing an immersive experience for onboard vehicle users. Tactile communication will also enhance specific vehicular applications such as remote driving, vehicle platooning, and driver training. The use of haptic-based warning signals can also help to improve driving safety. Tactile-based V2X can also benefit vulnerable road users by providing appropriate haptic signals that enhance their safety and activity. However, as noted in [26], there are several challenges in implementing this technology. Tactile communication requires high-speed, low-latency communication that can exchange large volumes of haptic information, which is difficult to achieve in high-mobility vehicular environments. Furthermore, the technology requires higher frequencies that are not very reliable, making it challenging to provide reliable high-rate communications. Other challenges include developing control and communication protocols, human-to-machine interfaces, haptic codecs, and the reconstruction of haptic data. The security of V2X networks [27] is essential for the large-scale distribution and verification of vehicular messages. To meet the security needs of different types of data, such as mission-critical messages and multimedia data services, different frame structures, routing strategies, and power/spectrum allocation methods are necessary.

Blockchain technology [4,28] is a potential solution to enhance the security and privacy of V2X communications. It can provide decentralized security management, mobile cloud/edge/fog computing, and content caching. A blockchain-based security solution can verify the authenticity of messages while preserving the sender’s privacy. Additionally, blockchain can be used for spectrum sharing, allowing different users to share the same spectrum, providing secure and highly efficient decentralized sharing. However, the adoption of existing blockchain technology is not feasible for V2X communication scenarios due to their dynamic network characteristics and real-time processing requirements. Developing new blockchain algorithms with ultra-low latency and addressing the limited throughput and scalability of current blockchain technology are significant challenges.

Quantum computing [29] is seen as a promising technology for 6G wireless communications, but it is still in its early stages of development. If practical solutions become available, quantum computing could enhance security in V2X communications, which is crucial for the prevention of accidents in autonomous vehicles. Traditional encryption strategies may not be adequate, and quantum computing’s inherent security feature of quantum entanglement makes it suitable to enhance security in V2X communications. Quantum computing can also offer an enhanced computational capability to optimize 6G-V2X services and execute complex optimization algorithms with reduced complexity. However, there are still many challenges to overcome, such as the development of large-scale quantum computing, the design of quantum security architectures, and the characterization of entanglement distribution. Current quantum computer chips can only operate at extremely low temperatures, making them difficult to use in vehicles, and significant research is needed on the thermal stability of quantum computer chips [30].

Recent advancements in machine learning (ML) research [31], aided by the availability of large datasets, high computational power, and storage capacity, have given rise to novel technologies such as voice assistants and self-driving vehicles. In the context of 6G vehicular networks [32], ML is an increasingly indispensable tool for highly autonomous and intelligent operation. Traditional wireless communication systems rely heavily on model-based approaches, which may not be accurate in certain scenarios, such as channel estimation and interference modeling. ML’s ability to extract features and identify hidden relationships between input and output data makes it a powerful tool in such scenarios where traditional communication system designs may fail. ML’s data-driven nature can also help to predict channel dynamics, network traffic, user behavior, application requirements, and security threats, leading to better resource provisioning and network operation [33]. In addition to enhancing road safety and driver experiences, advancements in ML techniques are contributing to the realization of autonomous cars. For instance, as seen in [34,35], data streams from cameras, GPS units, sensors, and LiDAR can be processed to make data-driven intelligent decisions through modular perception–planning–action or end-to-end learning methods. Thus, research focuses on the impact of ML on 6G-V2X networks, discussing the opportunities, challenges, and overall vision, with a focus on the physical layer, radio resource allocation, and system security. Additionally, researchers have introduced federated learning, one of the most promising ML technologies.

The objective of the research study in [36] was to optimize the gas solubility and forecast the traffic flow in vehicular networks enabled by 6G. The authors emphasize that gas solubility is an essential aspect of vehicular networks as it impacts the energy consumption, quality of service, and safety of vehicles. To optimize the gas solubility, the authors suggest a novel approach utilizing deep learning techniques. The deep learning model is trained on traffic flow data and can predict the ideal gas solubility for varying traffic scenarios. Additionally, the authors propose a traffic flow forecasting model that uses historical traffic data and deep learning techniques to forecast the traffic flow in real time. The anticipated traffic flow can subsequently be employed to optimize the gas solubility and enhance the overall performance of vehicular networks. The authors evaluate the proposed approach through simulations and experiments and observe that the traffic flow forecasting model can predict the traffic flow accurately. The optimized gas solubility improves the quality of service, energy consumption, and safety of vehicles in different traffic scenarios. Overall, the study suggests a novel deep learning-based approach for the optimization of gas solubility and prediction of traffic flow in 6G-enabled vehicular networks, which could enhance the efficiency and performance of vehicular networks and enable intelligent transportation systems in the future.

Stefano et al. [37] present a novel bidirectional visible light communication (VLC) system and evaluate its performance via various parameters, such as the data rate, latency, reliability, and coverage for 6G vehicular communication. The bidirectional VLC system outperforms the IEEE 802.11p and LTE/5G C-V2X technologies in terms of the data rate and latency. The bidirectional VLC system can achieve a data rate of up to 10 Gbps, which is significantly higher than that of the other technologies. Moreover, the VLC system has lower latency than the other technologies, which is important for safety-critical applications in 6G-enabled vehicular networks.

The protocols proposed by Pandi et al. [38] are designed to ensure secure communication in vehicular ad hoc networks (VANETs) enabled with 6G technology. The authors use a combination of batch authentication and key exchange techniques to improve the network efficiency and reduce the communication overhead. The authors add two new protocols for anonymous batch authentication and key exchange in VANETs enabled with 6G technology: ABKA and ABKA-R. ABKA allows multiple messages to be authenticated simultaneously, thereby reducing the communication overhead. ABKA-R is an improved version of ABKA that includes a revocation mechanism to handle compromised or malfunctioning nodes. The authors describe the message flow, security properties, and advantages of each protocol.

Jiayu and Gao [39] make certain assumptions about the network and its attackers, such as the availability of public key infrastructure (PKI) and the existence of malicious nodes in the network. The paper also defines the notations and terminologies used in the proposed protocols, including batch authentication, key exchange, and anonymity. Fan et al. [40] demonstrate that the proposed protocols provide strong security guarantees, including confidentiality, integrity, authenticity, and anonymity. The paper also evaluates the performance of the proposed protocols in terms of communication overhead, computation time, and memory usage. The results show that the proposed protocols are efficient and can be implemented in practice.

The main challenge for web service providers [41] is to provide internet access inside moving vehicles with the best speed possible. The cloud radio vehicular ad hoc network architecture is a sensible solution for data broadcasting from the cloud to moving vehicles within a specific radius for road safety and traffic supervision. Traditional radio network architectures [42] are less efficient with respect to energy consumption, so a multilayer system with the elimination of intermediate points is proposed to improve energy efficiency. The creation of a 6G environment with dynamic objects is possible by setting dedicated paths with higher speeds and energy efficiency. A neuro-fuzzy system is proposed [42,43] as an efficient tool to reduce intermediate points, and a comparison with various parameters is performed to validate its effectiveness. VANETs can benefit from the Cloud Radio Network (C-RAN), which provides a durable platform for ad hoc networks. Node selection can be improved with Learning Vector Quantization (LVQ) and neuro-fuzzy techniques such as back-propagation. Next-generation technology, such as a sixth-generation virtual network, may be introduced in the next decade using advanced techniques such as learning vector quantization and a neuro-fuzzy forwarder scheme.

The cost-effective interchange and viability of buses used as mRSUs [1] have been investigated, and the results show that the performance was improved after the sRSU was replaced by the mRSU, but the costs increased and remediation was difficult, so both static and moving RSUs were implemented in accordance with all relevant requirements: static, installed on public transportation, and mobile. The simulation results [44] corroborate the analytical model’s findings, which show that the greater the number of mRSUs, the greater the probability of communication in VANETs. According to the findings, adopting a 5 per cent rate in mRSUs may increase the communication efficiency by two times while decreasing the cost. Because the automobile is not under fixed RSU radio coverage, it may communicate with adjacent mRSUs to obtain traffic information. The simulation findings of the social-aware mobile roadside unit scheme [45] for content distribution in vehicular social networks show that it improves the source vehicle’s mean throughput performance. It is a cross-layer RSU/mobile relay station (RS) detection method for VANETs that takes into account many variables from various protocol levels, such as the connection status, bandwidth, and latency aspects, and user services.

## 3. Methodology

To improve the connection stability and to reduce the packet loss during the handover from one RSU to another, this research introduces the mRSU; in addition, to evaluate the results, the urban mobility traffic scenario is considered. For the simulation and results, the NS-3 simulator is selected, in which the scenario is designed, and then the results of the simulation are compared to other conditions in which the RSU is static. As in [46], the standard energy that is assumed for the storage, carrying, and transmission of the message of 500 KB is 1.7 J, but this is only the assumed value. Our results indicate the actual value at the node that is required to store, carry, and transmit the message. We then note the value that a mobile node consumes, while performing normal operations; after this, the sender sends a message and we note the change in energy consumption at the receiver node. The value that is finally found is 4.3136 J for a message of 0.16 KB.

To measure the energy and power consumption when sending or receiving a message, in this research, a DC power supply is used, which will change in current and voltage when sending or receiving a message. Thus, by obtaining the values of the current and voltage, the power can be determined; then, by obtaining the value of the power, the energy consumption is calculated. At the start, the DC power supply is used during the movement of the vehicle. Here, to determine the values for current and voltage, a DC power supply (Power Net 1502DD) of 15 V and 2 Amperes (A) is used.

**Figure 3:**
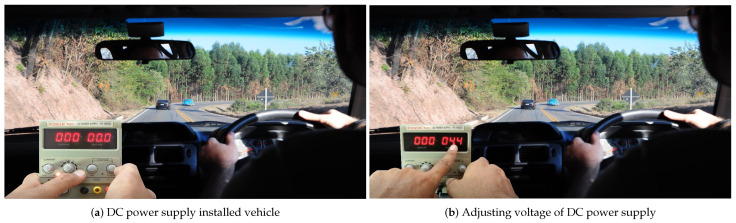
Evaluating experimental data.

**Figure 4:**
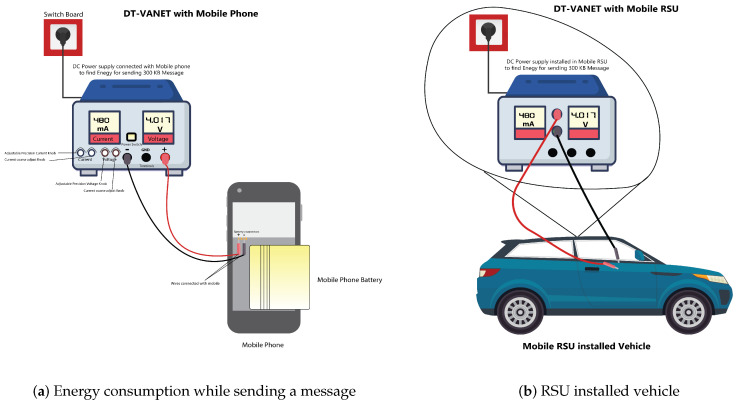
Computation of experimental data.

Initially, the voltage encoder wheel is set to 4.4 V because the nodes mostly bear a voltage of around 4.4 V and, after increasing this, there will be the possibility of IC damage; thus, we set the voltage at 4.4 V and the current to a maximum of 2 Ampere to calculate the power consumption of the mRSU before sending and receiving the message as shown in Figure 3. Next, we connect the positive terminal of the power supply with the positive terminal of the node and the negative one with the negative terminal. Next, before sending or receiving any message, we first observe on the screen that the mRSU is consuming a current of 480 mA at a voltage of 4.017 V; there is a small variation from the set value, so the power is calculated by using the product of the voltage and current, which is 1.92816 Watts before sending any message. Then, by maintaining the same motion at the speed of 15 km/h, we send a message of 300 KB to another node while keeping the DC power supply connected to our mRSU, and we note the readings. There is a change in value and the current value reaches 362 mA, while the voltage reaches 4.002 V, thus, the power value at this point is 1.449086 Watts as shown in Figure 4. Therefore, taking the difference between the two values of power, we determine the amount of power consumed when sending the message. We calculate this energy value using the change in the values of current and voltage, and we then calculate the power; from the power, the energy consumption can be calculated.

### 3.1. Proposed System Model

**Figure 5:**
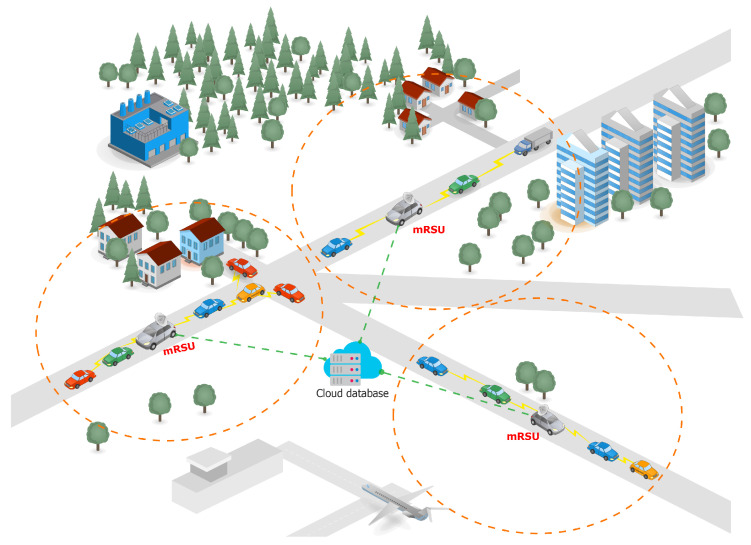
Scenario with mobile RSU.

**Figure 6:**
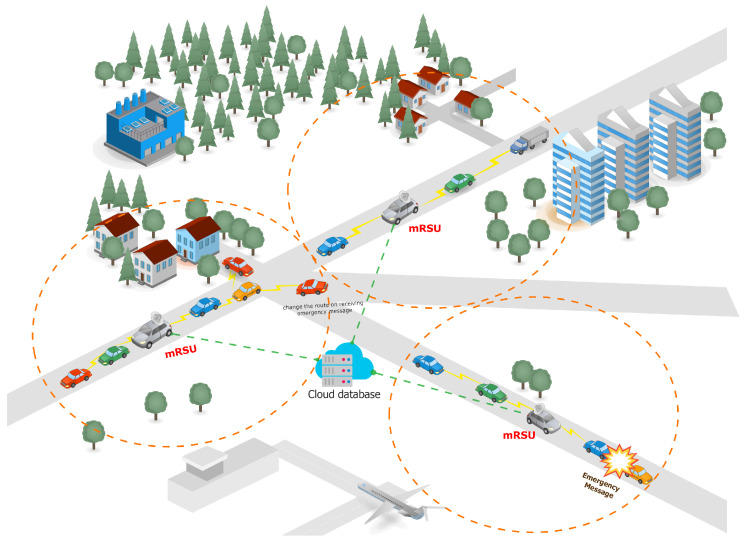
Urban scenario during accident.

Figure 5 and Figure 6 show the network topology and transmission protocols, respectively. This research solely analyzes the routing of downlink transmissions from RSUs to vehicles, because this is the main source of energy consumption in mRSUs. The specific energy preservation at the mRSU may then be determined. After calculating the change in voltage and current in a real-time scenario, the energy consumption can also be discovered using other parameters by simulation in the NS-3 for urban mobility with the mRSU in the VANET.

### 3.2. Communication Model

This research considers an urban smart city environment with a vehicular ad hoc network and mRSUs installed. The network is composed of a series of mRSUs with random, non-overlapping, and overlapping neighboring coverage zones. Each RSU is powered solely by a certain quantity of energy, which is gathered and stored in a finite-capacity storage medium. We characterize each mRSU’s storage device as a finite energy queue with capacity and a preserved quota for emergency messages.

### 3.3. Topology Model

Each mRSU employs a power system to broadcast a fixed number of bits in each time slot, regardless of the vehicle’s location within its communication range. For the sake of simplicity, this research presumes that each mRSU has a single channel to assign to a vehicle at any particular time and that each vehicle has a requirement of a certain number of bits that can be fulfilled in a single time slot. At first, we calculate the mRSU’s energy consumption when interacting with the vehicle during time slot ‘t’. We utilize the following model, since it is dependent on the location of the vehicle within the mRSU’s transmission range.

### 3.4. Experimental Values of Energy Consumption

Practically, this energy is calculated from the change in the values of current and voltage; thus, we calculate the power. Then, from the power, the energy consumption is calculated. When the message size is 0.16 KB, the energy consumption will be estimated to align with the ensuing figures

Before sending message

Current=441mA

Voltage=3.682V

P=V×I

P=3.682V×441mA

(1) $$P_{1}=1.623762\;W$$

Current=426mA

Voltage=3.685V

P=V×I

P=3.685V×426mA

(2) $$P_{2}=1.56981\;W$$

Thus, the change in power can be found by subtracting Equation (2) from Equation (1):

(3) $$P_{1}-P_{2}=1.623762-1.56981=0.05392\;W$$

Consequently, the power consumption during this message is =0.05392 W.

E=P×T

E=0.05392×40s

(4) E=2.1568J

Hence, for a 0.16 KB message size, the energy consumption is 2.1568 J.

Here, the packet size is in KB, the current is in mA, and the voltage value of the node is in volts. To calculate the power consumption and energy consumption for message transfer, we use the formula and multiply the voltage and current to obtain the power in Watts. As in physics, the power is the amount of energy transferred per unit of time. Here, to find the energy, the power is multiplied by the time and we obtain the result in J, which is the energy consumption during the message transfer, i.e., 2.1568 J. In the actual scenario, the message size is about 300 KB, and we have to determine the energy consumption for a 300 KB message. For this, there are 2 methodologies: we can calculate it using the unitary method or by practically sending a message of 300 KB and checking the change in energy consumption at the receiver end. We implement both methodologies and obtain the following results. When implementing the unitary method, the value is about 4.044 kJ for a 300 KB message; meanwhile, when calculating the real-time value by sending the message from one node to another, the change in energy at the receiving end is calculated using the Ampere Meter app and is 0.5546202 J for a 300 KB message. We can calculate the energy consumption by applying the “unitary method” for a 300 KB message size as follows.

Energy consumption for 0.16 KB message is 2.1568 J.

Energy consumption for 1 KB message is 2.1568/0.16 = 13.48 J.

Energy consumption for 300 KB message is 13.48 × 300 = 4.044 kJ.

By applying the unitary method, we calculate the energy consumption for the 300 KB message and it is 4.044 kJ.

The experimental energy consumption values for a 300 KB message size are shown in Table 1.

Average energy consumption for 300 KB message = 30.14563 J.

As there is a large difference between the values, we apply the unitary method at the nodes, so the real-time value is more applicable. This research will proceed with “30.14563 J for 300 KB”. From the above table, one can see small variations in the energy values when changing the speed and distance of the nodes. In this case, upon increasing the speed or distance, in both situations, the value of energy consumption or power applied is increased. At this point, we assume the average value of time as 40 s for all cases; however, for the change in distance and speed, it may vary too. As the RSU is mobile and thus consumes energy while sending and receiving messages, in this case, on average, if an mRSU is sending a message 15 times per day, then the average energy required by a single mRSU is about 324 J. If this energy is reserved specifically for message transmission and the rest can be used for other functions, message transmission will not be disturbed, during when there is very little energy available.

Average energy required for one day = 324 J.

For the simulation scenario, this research will consider random vehicles having an mRSU in motion and will calculate the energy consumption while sending a packet from the mRSU to the vehicle. As both the vehicular nodes and the RSU are in motion, considering the urban city environment, we have an area of 2000 m${}^{2}$. At first, we consider the AODV protocol for the simulation, using the two-ray ground propagation model with the initial energy of 50 J and a total simulation time of 150 s. Similarly, while the simulation varies the speed of the mRSU, we changes the protocol and vary the topology and movement to analyze the effects on the parameters, including the energy consumption of the RSU, packet delivery ratio, hop count, average delay, and average throughput.

### 3.5. Simulation Environment

**Figure 7:**
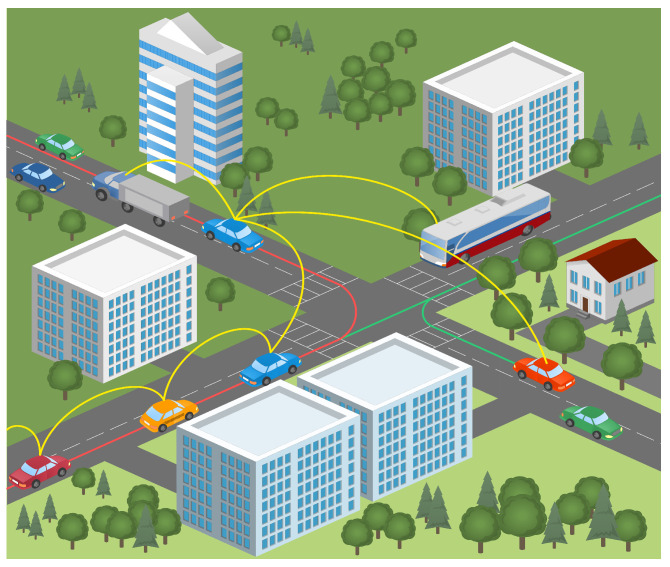
Intelligent transportation system (ITS).

The simulations were carried out using Simulation of Urban Mobility (SUMO) [47], employing realistic scenarios exported from the Open Street Maps (OSM) [48] tool. The trace files created by SUMO are utilized in NS-3 [49] for additional simulation. The simulation results are provided and thoroughly examined. In the high-mobility VANET environment, an effective routing system is required for appropriate data transmission. Topology-based, location-based, reactive-based, proactive-based, cluster-based, and opportunistic-based are some of the routing protocols that have been suggested in the past. This research examines the performance of the AODV [50] reactive protocol for both the mRSU and the sRSU in a workable VANET scenario. Additionally, route loss owing to impediments (such as cars, buildings, road curvature, and so on) along the path of signal transmission from one vehicle to another must be considered, as shown in Figure 7.

Data packet loss reflects the network channel properties, while route loss is estimated using propagation models. As a result, route loss and fading models, as well as correct routing protocol selection, are examined. The IEEE 802.11p and IEEE 1609.14 standards are included in the WAVE. CSMA/CA [51] is implemented in the IEEE standard with QoS and reliable communication. As a result, interface protocols such as IEEE 802.11p and 1609.x allow effective short-range transmission in this context.

At each channel, different traffic is generated and we analyze the energy consumption for each node. In this scenario, there is a “tcp” agent that is integrated with n0 as well as n2, and communication takes place to a “TCP” “sink” agent connected to n1 and n3. The maximum size of a packet created by a “TCP” agent is 1 KByte by default. A tcp “sink” agent produces and transmits ACK packets to the sender (tcp agent), and then frees the packets that it has obtained. A “UDP” agent connected to n4 and n5 is linked to a “null” agent connected to n6 and n7, respectively. A “null” agent simply frees the packets that have been received. To the “TCP” and “UDP” agents, an “FTP” and a “CBR” traffic generator are connected, respectively. The “CBR” is configured to produce 1 KByte packets at a 1 Mbps rate. “CBR” is planned to begin and stop, while “FTP” is set to begin at 1.0 s and end at 15.0 s.

Here, at the start, the NS-3 script generates an object instance and assigns it to a variable; then, we initialize the packet format. After this, we create a scheduler and then select the default address format. The simulator object has member functions that create compound objects such as nodes and links; it then connects the network component objects created. After this, it sets the network component parameters and then creates connections between agents (forming a connection between “tcp” and “sink”). Similarly, the “Cell Breathing” concept of the varying coverage area of mRSU will be considered, as it appears in UMTS. According to this, the coverage area will be increased where the traffic travels only in one direction or is not too abundant, while the coverage area of the mRSU will be decreased to provide the maximum connectivity among vehicles in the coverage of a relative mRSU.

### 3.6. Edge Computing in VANETs

Edge computing is a paradigm within distributed computing that enables the processing of data at the edge of the network, closer to the source of the data. It aims to reduce the latency and bandwidth requirements associated with sending data to the cloud for processing, while providing real-time data analysis and responses. Research is being conducted to develop architectures for edge computing that can efficiently process data closer to the source. This includes investigating various edge computing models, such as cloud edge computing, fog computing, and mobile edge computing. It has a wide range of potential applications, including industrial Internet of Things (IoTs), smart cities, healthcare, and autonomous vehicles. Research is focused on developing applications that can benefit from edge computing and designing solutions that address the specific requirements of these applications. As edge computing involves processing data closer to the source, security is a major concern. Thus, it is necessary to develop security solutions that can protect edge computing devices and networks from various threats, such as data breaches, malware attacks, and denial-of-service attacks. It also requires a robust and reliable network infrastructure to enable efficient communication between edge devices and the cloud. Research in [18] focused on developing networking solutions that can support edge computing, such as 5G, SDN, and NFV. Edge computing involves processing data in real time, which requires high performance and low latency. Research is also focused on optimizing the performance of edge computing systems, including developing efficient algorithms, optimizing resource allocation, and reducing energy consumption. By processing data at the edge, edge computing can significantly reduce the amount of data that need to be transmitted to the cloud. This can help to reduce the bandwidth requirements and associated costs. Edge computing [28] can enable new business models, such as providing edge services to customers or leveraging edge computing to enable new products or services. For example, edge computing could be used to provide real-time monitoring and analysis for industrial IoT applications. This may include developing new algorithms and analytics tools, as well as building applications that can operate effectively in resource-constrained edge environments. Edge computing research is focused on developing technologies and architectures for the efficient and effective processing of data at the edge of the network.

Mobile edge computing (MEC) [7] is a paradigm within distributed computing that enables the processing of data at the edge of the mobile network, closer to the end-user devices. Research is focused on developing architectures for MEC that can efficiently process data closer to the end-user devices. This includes investigating various MEC models, such as centralized MEC, decentralized MEC, and hybrid MEC.

**Figure 8:**
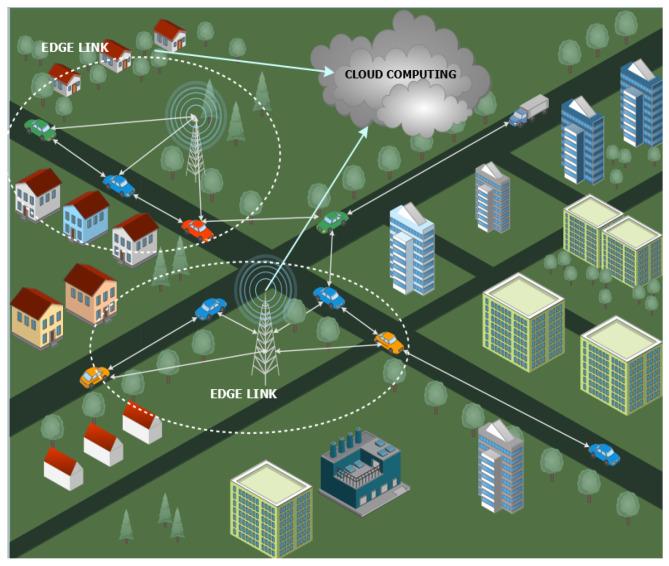
Edge computing.

Edge computing can also improve the reliability of communication in VANETs by reducing the dependence on centralized servers. By distributing computing resources and services closer to the source, edge computing [40] ensures that communication can continue even in the event of a network outage or server failure. This is particularly important in the context of VANETs, where communication can be disrupted by factors such as high-speed mobility, signal attenuation, and interference. Edge computing can also enhance privacy and security in VANETs by reducing the need to transmit sensitive data over the network, as shown in Figure 8. By processing data locally, edge computing can limit the amount of data that need to be transmitted over the network, which can reduce the risk of data interception and unauthorized access. Edge computing can also enable the use of encryption and other security measures closer to the source, which can further enhance security. It can also improve the utilization of computing resources in VANETs by distributing computation and storage tasks to devices in the network. This can reduce the load on centralized servers and enable the more efficient use of available resources. Edge computing can also enable the use of low-power devices, such as smartphones and tablets, as computing nodes, which can further enhance resource utilization.

### 3.7. Routing Protocol

Because of the increasingly innovative layout, diverse network features, and many forms of communication, effective routing is difficult (i.e., V2V and V2I). The constant mobility of vehicles needs reliable network connectivity. An effective routing protocol must offer quality of service (QoS), improved traffic and network management, mobility, and delay-tolerant message delivery as a top priority. The protocol under consideration is topology-based, with routing data mostly kept in the routing domain. For good routing decisions from transmitter to recipient through a sufficient transceiver, an efficient routing system is required.

### 3.8. Ad Hoc On-Demand Distance Vector (AODV)

An ad hoc on-demand distance vector (AODV) is a routing algorithm with a traditional routing table based on the concept of the distance vector algorithm [50]. The node is deleted from the table if the network is not completed within a certain amount of time. All data and communications from neighbors are carried via the supplier and intermediary nodes. For connectivity, a control message is forwarded, and a path is identified by delivering a route request (RREQ) message.

### 3.9. Interfacing Protocol

The IEEE 802.11p PHY/MAC protocol with multichannel expansions based on the IEEE 1609 WAVE standard is used in this investigation.

### 3.10. IEEE 802.11p

IEEE 802.11p and 802.11b [29] are commonly utilized for wireless connections in vehicle communication. IEEE 802.11p is a PHY/MAC cross-layer standard that incorporates OFDMA [31] and uses a frequency spectrum of 5.9 GHz for V2V communication. The IEEE 802.11p MAC is designed on CSMA/CA, which stands for Carrier Sense Multiple Access with Collision Avoidance. The control channel (CCH) is devoted to broadcasting, while the service channel (SCH) [50] is used for non-safety transmissions, as a component of the multichannel access process. Before the broadcast, a node detects the channel, and, throughout transmission, the node and channel become dormant for a short inter-frame space (SIFS) [51]. Only when the channel is empty does broadcasting restart. In the event of a busy channel, an adjustable back-off scheme is developed using a contention window and only transmits when the back-off timer has expired. Control packets are transmitted across the CCH with no requirement for the receiver to acknowledge them. If no acknowledgement is received within the time interval, the packets are repeated in SCH.

### 3.11. IEEE 1609

The protocol incorporates communication and operational requirements, as well as information security and management, to allow multichannel functioning. IEEE 1609 [52] is composed of four standards: IEEE 1609.1 for application development, IEEE 1609.2 for privacy and protection measures, IEEE 1609.3 for WAVE managerial and networking services, and IEEE 1609.4 for multichannel processes with logical connections and rules at every level.

### 3.12. Performance Evaluation Metrics

The evaluation criteria used in the comparison are listed below. Table 2 summarizes the terms that were utilized.

### 3.13. Simulation Environment and Parameter Setup

The efficiency of V2I and V2V connections may be improved by using efficient routing techniques and propagation algorithms. Routing methods and propagation designs are evaluated in terms of throughput, average delay, packet delivery ratio, average routing throughput, and MAC/PHY overhead using large numbers of nodes and simulated time frames. The parameter settings for the simulation scenario are shown in Table 3.

### 3.14. Generation of Simulation Scenario

**Figure 9:**
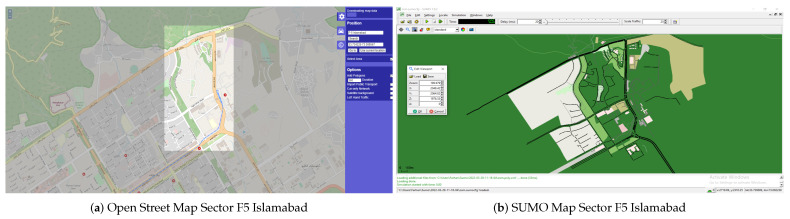
Computational framework.

The reference point is obtained from the OSM, which depicts a realistic setting of Islamabad, Pakistan’s capital (Sector F-5). The .osm file is fed into SUMO, which generates the mobility.tcl file, which contains information about every node (vehicle), such as the number of vehicles and their positions, speeds, and directions. The mobility.tcl file is used to run the simulation in NS-3. The 802.11p MAC/PHY, with a frequency of 5.9 GHz, is kept steady for the delivery of basic messages to analyze the surrounding BSM. Variations in transmission schemes and routing protocols depending on the simulation time and the number of nodes are used to evaluate the performance of the network (vehicles). Figure 9 shows a screenshot of a plausible scenario with the simulated SUMO scenario generated.

### 3.15. Propagation Models for Simulation

In V2V and V2I communication, propagation models are fundamental in defining the channel properties. Path loss and signal fading are two propagation parameters that alter the signal intensity as it travels from the source to the destination in a wireless medium. The Friis path loss/FreeSpace, two-ray ground, log distance path loss, Nakagami, Rician, Rayleigh, range propagation, and shadowing models are all used in this study.

#### 3.15.1. Friis Propagation Model

Friis is among the earliest propagation models introduced for wireless networks [50], in which radio signals propagate in an omnidirectional manner from the source. The Friis propagation loss model is applied in this scenario. The following is the actual mathematical expression:

(5) $$\frac{P_{r}}{P_{t}}=\frac{A_{r}A_{t}}{d^{2}\lambda^{2}}$$

For the situation of an isotropic antenna with no heat loss, we use the following mathematical statement:

(6) $$A_{isotr}=\frac{\lambda^{2}}{4\pi }$$

The final equation is thus

(7) $$\frac{P_{r}}{P_{t}}=\frac{\lambda^{2}}{{\left(4\pi d\right)}^{2}}$$

The radio waves travel through redundant space, and the power received is calculated using the power broadcast, the gain of the antenna, and the distance between the sender and receiver [50]. This system is based on the transmitter’s broadcast range. The message is collected if the recipient is within the transmitter’s reach; alternatively, it is dropped.

(8) $$P_{r}=\frac{P_{t}G_{t}G_{T}\lambda^{2}}{{\left(4\pi d\right)}^{2}L}$$

The transmission power (W) is $P_{t}$, the reception power (W) is $P_{r}$, and the wavelength is (m). $G_{t}$ and $G_{r}$ are the transmission and reception gains, respectively. The network loss is *L* and the transmission distance and recipient are *d*. The received radio signal intensity is determined by the broadcast signal intensity, the reception and broadcasting antenna gain, and the separation between the transmitters to the receiver, according to the equation mentioned above.

#### 3.15.2. Three-Log Distance Propagation Model

This approach works well in densely populated areas. The model calculates the route loss across a greater distance and range of communication [29]. This model calculates the route loss caused by the distance between a transmitter and a receiver. The model assumes an exponential path loss across the distance between the transmitter and receiver.

(9) $$L=L_{o}+10n\log(\frac{d}{d_{o}})\;$$

The path loss is designated by *L*, the length is indicated by d, and the reference distance is represented by $d_{o}$; the path loss for the reference distance is defined as $L_{o}$, and the path loss exponential is marked by *n*.

#### 3.15.3. Nakagami Fading Model

The Nakagami model essentially a fading model that accounts for multi-path fading-induced signal intensity variations. Because the model is not well suited for simulations as a route loss model, it can be combined with other loss models to improve the performance [29]. It has a gamma distribution and a continuous probability distribution for various settings.

(10) $$p\left(x;m,\;\omega \right)=\frac{{2m}^{m}}{\Gamma \left(m\right)\omega^{m}\;}\;x^{2m-1}\;e^{-\frac{m}{\omega }x^{2}}$$

The fading depth and average power received are denoted by the *m* and ω variables, respectively. Updating the gamma distributions yields the probability density function (pdf) at a given distance. As with a stochastic model, the message continues to follow a gamma distribution with average power fading and intensity with amplitude x≥0. When m=1, the Nakagami model transforms into the Rayleigh model.

#### 3.15.4. Rician Fading Model

Rician fading is a stochastic process for radio signal propagation anomalies induced by partial message suppression. Multi-path interference occurs when a signal reaches the recipient through two separate pathways, at most one of which is altering (prolongation or decreasing) [51]. Whenever one of the pathways, usually a line-of-sight signal, is considerably stronger than most others, Rician fading occurs. A significant dominating feature is found in Rician fading. The in-phase and quadrature-phase components of the received signal are simultaneously Gaussian random variables, comparable to Rayleigh fading. Owing to a determinate powerful waveform, the average rate for (at minimum) a single part is non-zero in Rician fading. A Rician distribution describes the amplitude gain at the reception. With parameter K, the Rician amplitude r can be expressed as

(11) $$r=\sqrt{{\left(\sigma x_{1}+A\right)}^{2}+\sigma x_{2}^{2}}$$

where $x_{1}$ and $x_{1}$ are quadrature values, while $A^{2}/2\sigma^{2}≅K,\;\sigma x_{i}$ is a random variable along with the variance $\sigma^{2}$. The mean-squared value of the Rician distribution is expressed by

(12) $$A^{2}+2\sigma^{2}=2\sigma^{2}\left(K+1\right)$$

and the normalized power envelope is derived from the previous two equations as

(13) $$\frac{r^{2}}{P}=\frac{1}{2\left(K+1\right)}\left[{\left(x_{1}+\sqrt{2K}\right)}^{2}+x_{2}^{2}\right]$$

*P* is the power in the dominating path as expressed by a large-scale model. The outcome of a sizable propagation model may be modulated with this power envelope. The power anticipated by the large-scale scenario is assumed to be the mean-squared value of the envelope. This power includes the dominant-path and multi-path power in the Rician model. In some transmission schemes, representing simply the value in the dominant channel will be much more suitable for large-scale power computation. The above equation is divided by $A^{2}=2\sigma^{2}K$. The normalized power envelope then becomes

(14) $$\frac{r^{2}}{P}=\frac{1}{2\left(K\right)}\left[{\left(x_{1}+\sqrt{2K}\right)}^{2}+x_{2}^{2}\right]$$

where *P* is the power in the dominating route as represented by such a large-scale model.

#### 3.15.5. Rayleigh Fading Model

**Figure 10:**
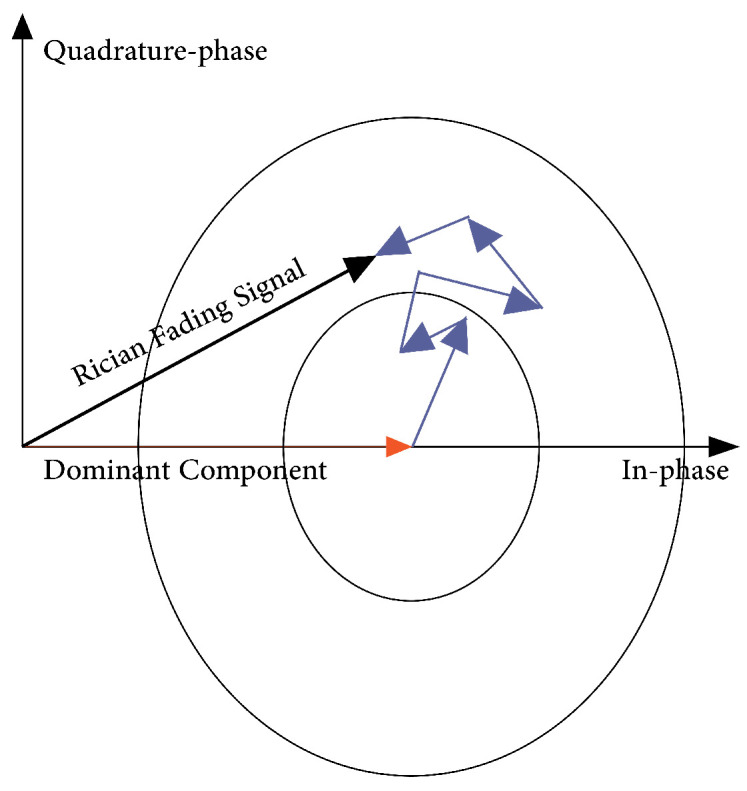
Rayleigh and Rician fading.

If there is no LOS and we have only multi-path features, the Rayleigh propagation model as shown in Figure 10 is used to simulate the scenario. Because several pathways can be combined, productively or ineffectively, this model encompasses extensive changes in received message strength [51]. The surroundings have a major impact on the amplitude, delay, and phase shift of these variables. A deterministic model in which a particular alteration is implemented is also required by the Rayleigh model.

(15) $$P_{r_{Rayleigh}}\left(d\right)∼Rayleigh\left(P_{r_{det}}\left(d\right)\right)$$

This Equation (15) can also be transformed with greater detail as follows:

(16) $$P_{r}\left(d\right)=P_{r_{det}}\left(d_{o}\right)\times 10^{PL\left(d\right)}\log\left(1-unif\left(0,1\right)\right)$$

Here, the power loss factor is represented as

(17) $$PL\left(d\right)=-\alpha log10\left(\frac{d}{d_{o}}\right)$$

#### 3.15.6. Range Propagation Model

The location (ranging) between transmission and reception determines the propagation loss. Fading is determined by the sole MaxRange property. The signal is received at the transmit level of power by the receiver at or within the MaxRange in meters. Beyond the MaxRange, the recipient obtains a strength of −1000 dBm (effectively zero). The size of this type is 48 bytes.

### 3.16. Evaluating Parameters of Simulation

#### 3.16.1. Average Throughput

The level of effective packet delivery via a communication connection is known as throughput. The sum of bits transferred over a connection per amount of measure or the number of received packets is divided by the final packets received minus the very first transmitted packet in this calculation [52]. The number 8 in the equation denotes the number of packets received and translated to bits. It is commonly expressed in bits per second (bps), kilobits per second (Kbps), or megabits per second (Mbps). The higher the throughput, the greater the network performance.

(18) $${Th}_{avg}=\frac{{(P}_{r\;}*8)}{{(T}_{l})-{(T}_{f})}$$

#### 3.16.2. End-To-End Delay

The overall time required for a message to travel from the transmitter to the recipient vehicle is computed as the total of all delays in the connection induced by intermediary nodes [52]. It is calculated by adding all transmission delays together. It is measured in seconds, milliseconds, or nanoseconds. A lower delay indicates the improved performance of the network.

(19) $$EED=\frac{\left(T_{delay}+P_{delay}+Q_{delay}+{Pro}_{delay}\right)}{totalP_{r\;}}$$

#### 3.16.3. Packet Delivery Ratio

The ratio of packages being sent by the sending vehicle to those collected through other vehicles is the packet delivery ratio (PDR). PDR ratings that are higher suggest better performance of the network.

(20) $$PDR=\frac{{totalP}_{r\;}}{totalP_{s}}$$

#### 3.16.4. Energy Consumption

The downlink connection between the mRSU and the vehicle is considered in this study utilizing only one channel. We suppose that the time is split into equal-length time intervals τ. The mRSU assigns each time slot to a vehicle for communication at a consistent data rate, independent of the vehicle’s location. As a result, the mRSU-to-vehicle link is subjected to power regulation [53]. Any vehicle that arrives transmits to the mRSU its current location and speed information. The cost of communication energy may be approximated based on the location of each vehicle. As a result, the mRSU distributes time slots to each node in such a manner that their energy usage is minimized [53]. Let $E\left(i,t\right)$ denote the energy usage for communication between the RSU and a vehicle *i* at time t, as calculated using a simplified path loss, and be equal to

(21) $$E\left(i,t\right)=P_{tx}\left(t\right)\tau =\frac{P_{rx}\left(i,t\right)\tau \;}{P_{o}\left[\frac{d_{o}}{d_{\left(i,t\right)}}\right]\gamma \;}=\frac{N\;(2^{\frac{D}{B}}-1)\tau }{P_{o}\left[\frac{d_{o}}{d_{\left(i,t\right)}}\right]\gamma }$$

Thus, in this equation, $P_{tx}\left(t\right)$ is the power transfer of the mRSU at the particular time slot *t*, while the received power of vehicle i is $P_{rx}\left(i,t\right)$, the reference distance is denoted by $d_{o}$, $P_{o}$ is the path loss at the reference distance, [54] the distance between the mRSU and vehicle *i* at time slot *i* is expressed by $d_{\left(i,t\right)}$, the path loss exponent is γ, D is the data rate, B is the bandwidth of the channel, and N is the noise power.

#### 3.16.5. Hop Count

The hop count is a parameter representing the number of distinct hops between multiple vehicles from the sender to the receiver inside a node radius. This is a measure of distance rather than the hop distance, because the hop distance, according to analysis, refers to the transmission distance for only a hop [55,56]. The bits, on either side, are passed from one hop to the next, with the computation cost depending on the distance between the transmitter and the receiver. The hop count is divided into two types: one is between the source node and the target node (next-hop node), and the other is between the next-hop node and the destination node. For the first category, the hop count is calculated using the node broadcast range, which is the node diameter. The initial hop count would be the neighbor node with the greatest distance from the source node within the node radius of X meters. The hops’ region will be limited to the propagation range of the node alone. The estimated hop count (*H*) may be used to acquire the farthest distance inside the node diameter.

(22) $$H=\frac{D}{l+d}$$

where

*D* = distance between a source and a target node;
*l* = average Length of a vehicle;
*d* = average distance between vehicles.

## 4. Results and Discussion

### 4.1. Results after Varying the Packet Size

The simulation was performed with various simulation durations and numbers of nodes for propagation channels and routing protocols across the IEEE 802.11p stack. The given routing algorithms and transmission models are evaluated, and evaluation metrics such as the packet delivery ratio, throughput, latency, hop count, and energy usage are addressed, predicated on the variable number of nodes and packet size. The following are some situations used to analyze the performance of the network.

#### 4.1.1. Simulation Results of Friis Propagation Model regarding Packet Size

The graph findings reveal that under the Friis propagation model, the mobile RSU outperforms the sRSU. Most notably, the mRSU performs well in terms of average latency since the receiving power is inversely proportional to the distance, and since the mRSU remains in movement with the vehicles, the length does not grow rapidly and the delay remains minimal. Consequently, as the packet size grows, the packet delivery ratio greatly increases and eventually becomes superior to that of the sRSU. Most importantly, in the Friis model or FreeSpace model, the energy consumption for the mRSU is the lowest, implying that by retaining the lowest energy quota, we may propagate far more data. The reason for this is that within the Friis model, power is inversely proportional to distance, and in the mRSU, the spacing among nodes and RSUs is smaller than in the sRSU; hence, less power is needed and much less energy is spent.

#### 4.1.2. Simulation Results of Three-Log Distance Propagation Model regarding Packet Size

In the three-log distance propagation model, the mobile RSU has a much smaller delay than the sRSU because the distance is limited; hence, the mRSU packet arrives at the node earlier. The mobile RSU also has a better packet delivery ratio because the possibility of packet dropping is reduced. After all, the RSU is in motion with the vehicles.

#### 4.1.3. Simulation Results of Nakagami Propagation Model regarding Packet Size

In the Nakagami propagation model, the mRSU surpasses the Nakagami model, notably in terms of the throughput and packet delivery ratio, because the multi-path process allows for numerous inputs at the receiver and higher throughput. Moreover, the capacity to receive numerous route signals minimizes the likelihood of packet drops.

#### 4.1.4. Simulation Results of Rician Propagation Model regarding Packet Size

Because Rician belongs to a multi-path propagation channel, the throughput of the mRSU is better here as well. Additionally, the average latency of the mRSU is reduced in the Rician propagation model as compared to the sRSU. Because the mRSU is in motion with the nodes, the distance between them is reduced, and therefore the latency is reduced. Similarly, the throughput increases owing to the multi-path process since messages can be received from any hop.

#### 4.1.5. Simulation Results of Rayleigh Propagation Model regarding Packet Size

The Rayleigh propagation model is a multi-path propagation channel; hence, the mRSU throughput is better here. Additionally, the average latency of the mRSU is shorter in the Rician propagation model as compared to the sRSU. Because the mRSU is in motion with the nodes, the distance between them is reduced, and therefore the latency is reduced. Similarly, the throughput increases owing to the multi-path process since messages can be received from any hop.

#### 4.1.6. Simulation Results of Range Propagation Model regarding Packet Size

The mRSU shows better performance in terms of the packet delivery ratio in the range propagation model since the range propagation model is based on the principle of delivering specific power in a specific range, so the probability of packet reception is higher in the range propagation model, especially in the mRSU, where a certain RSU moves together with the nodes.

### 4.2. Discussion of mRSU and sRSU for Propagation Models regarding Packet Size

**Figure 11:**
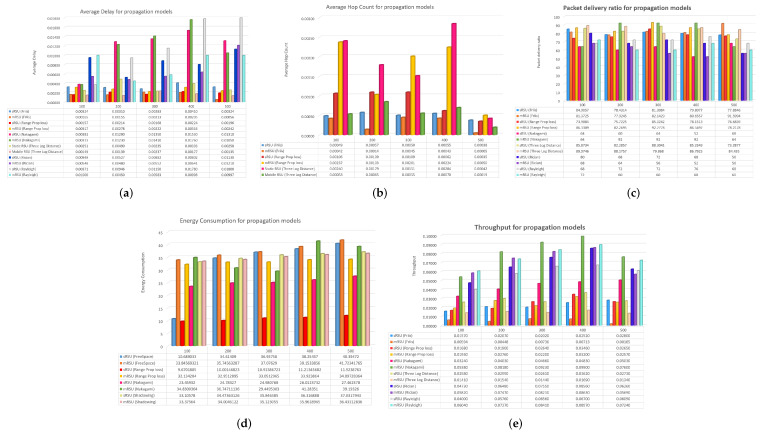
Computational modeling outcomes pertaining to the deployment of mRSUs and sRSUs in the context of propagation models, in relation to the variation in the packet size. (**a**) Average delay analysis of mRSU and sRSU with regard to packet size; (**b**) hop count analysis of mRSU and sRSU with regard to packet size; (**c**) PDR analysis of mRSU and sRSU with regard to packet size; (**d**) energy consumption analysis of mRSU and sRSU with regard to packet size; (**e**) throughput analysis of mRSU and sRSU with regard to packet size.

Regarding constant parameters, this instance takes Sector F5 in Islamabad, Pakistan, with routing algorithm AODV and a simulation time of 360 s, vehicle velocity of 20 m/s, and the random way point mobility model. Every node transmits power and the broadcast range is similarly set. The performance characteristics of the propagation model are evaluated with the packet size modifications. Figure 11a shows that the Friis model and three-log distance model outperform the other models both for the mobile RSU and sRSU in terms of end-to-end delay, because the receiving power is inversely proportional to the distance, and as the mRSU remains in motion with the vehicles, the distance does not increase rapidly and the delay remains the lowest. Meanwhile, the Nakagami model gives the worst result regarding the delay due to the variations in signal strength due to multi-path fading.

For the packet delivery ratio, the mRSU gives the best results at all packet sizes in the different models, e.g., at 100 KB, the mRSU in the range propagation and three-log models gives the best results, while, at 200 KB, 300 KB, and 400 KB, the mRSU in the Nakagami model gives the best results, and at 500 KB, the Friis model gives the best result. Overall, the mRSU gives best packet delivery ratio at all packet sizes because the possibility of packet drop reduces as the RSU is in motion along with the vehicles. On the other hand, the throughput, as shown in Figure 11e, shows better performance with the increase in the packet size for the mRSU in the Nakagami, Rician, and Rayleigh propagation models and shows a gradual increment. Thus, the throughput presents better results for the mRSU, especially for Nakagami, because, through the multi-path process, the MIMO helps to receive multiple inputs at the receiver and achieves greater throughput. On the other hand, regarding the energy consumption, the sRSU consumes less energy than the mRSU in all propagation models because of its continuous mobility; however, it is necessary to determine the quantity of energy in the mRSU that should be reserved for emergency packets per day. Overall, the mRSU outperforms the sRSU in terms of the throughput, end-to-end delay, and packet delivery ratio.

### 4.3. Results after Varying the Number of Vehicles

#### 4.3.1. Simulation Results of Friis Propagation Model regarding Number of Vehicles

The mRSU outperforms sRSU in the Friis propagation model. Since the reception power is inversely proportional to the distance between the mRSU and the node, the mRSU has the lowest delay at all points on the x-axis. As the mRSU remains in movement with the vehicles, the length does not rise substantially, and the delay remains minimal, while the throughput of the mobile RSU increases as the number of vehicles rises and the throughput of the static RSU gradually decreases. In the vast majority of situations, the packet delivery ratio of the mRSU is the best.

#### 4.3.2. Simulation Results of Three-Log Distance Propagation Model regarding Number of Vehicles

In the three-log distance propagation model, the mRSU has a smaller average delay than the sRSU at all x-axis points because the spacing is limited in the three-log distance or log distance propagation model; hence, the mRSU packet is obtained at the node relatively early. For the packet delivery ratio, the mRSU also gives good outcomes because the probability of packet loss decreases as the RSU is in motion together with the vehicles.

#### 4.3.3. Simulation Results of Nakagami Propagation Model regarding Number of Vehicles

The mRSU in the Nakagami propagation model operates well in terms of throughput but performs poorly in terms of average latency owing to multi-path fading. However, due to the MIMO feature of the Nakagami propagation model, the packet delivery ratio of the mobile RSU grows as the number of vehicles rises, while the PDR of the static RSU drops as the number of nodes increases.

#### 4.3.4. Simulation Results of Rician Propagation Model regarding Number of Vehicles

Due to its association with a multi-path propagation channel, the mRSU demonstrates superior performance in terms of throughput in the Rician scenario. Furthermore, when compared to the sRSU, the average latency is decreased in the Rician propagation model for the mRSU. This reduction in latency is a result of the mRSU being in motion alongside the nodes, leading to a decrease in the distance between them. Consequently, the latency is reduced. Additionally, the multi-path process enhances the throughput by enabling the acceptance of messages from multiple hops.

#### 4.3.5. Simulation Results of Rayleigh Propagation Model regarding Number of Vehicles

The Rayleigh propagation model is a multi-path propagation channel; hence, the mRSU throughput appears to be better here. Additionally, the average latency of the mRSU is shorter in the Rician propagation model as compared to the sRSU. Because the mRSU is in motion with the nodes, the space between them is reduced, and therefore the latency is reduced. Furthermore, the throughput increases owing to the multi-path method since messages can be delivered from any of the hops.

#### 4.3.6. Simulation Results of Range Propagation Model regarding Number of Vehicles

The mRSU appears to be better in terms of the packet delivery ratio in the range propagation model; because the range propagation model is based on the premise of conveying specific power in a specific range, the probability of packet reception is higher, especially in the mRSU, where the RSU is moving beside the nodes.

### 4.4. Discussion of mRSU and sRSU for Propagation Models regarding Number of Vehicles

**Figure 12:**
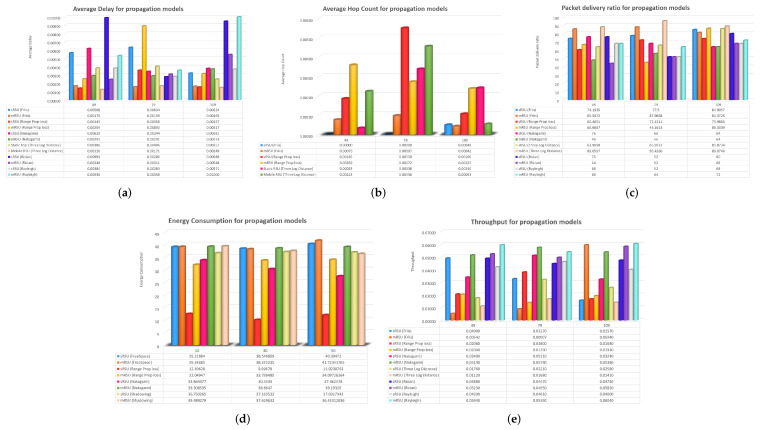
Computational modeling outcomes pertaining to the deployment of mRSUs and sRSUs in the context of propagation models, in relation to the variation in the number of vehicles. (**a**) Average delay analysis of mRSU and sRSU with regard to number of vehicles; (**b**) hop count analysis of mRSU and sRSU with regard to number of vehicles; (**c**) PDR analysis of mRSU and sRSU with regard to number of vehicles; (**d**) energy consumption analysis of mRSU and sRSU with regard to number of vehicles; (**e**) throughput analysis of mRSU and sRSU with regard to number of vehicles.

The propagation models using a consistent routing protocol, an interfacing protocol, and an increasing number of nodes are evaluated in this scenario. A realistic situation is used, with 49, 79, and 109 vehicles interacting through 500 KB packets. The mRSU in the three-log distance and Friis propagation models has the best performance since it has the least latency, as seen in Figure 12a. In the event of a delay, the sRSU and mRSU for the Nakagami propagation model operate similarly. The highest packet delivery ratio is shown in Figure 12c using the mRSU for the Nakagami, Rician, and Rayleigh propagation models.

However, the throughput of the sRSU in the Friis propagation model decreases as the number of nodes increases, but the throughput of the mRSU rises as the number of nodes increases. For the mRSU, the packet delivery ratios in the Friis, three-log distance, and Rayleigh propagation models show the best outcomes, but the performance of the mRSU in Nakagami improves dramatically as the number of nodes increases, while the sRSU delivers the opposite results. In terms of energy consumption, when the number of nodes is varied, the FreeSpace and shadowing propagation models produce nearly identical performance for both sRSUs and mRSUs, whereas the sRSU’s energy consumption is lower than that of the mRSU in the range propagation and Nakagami propagation models. However, the precise energy quota to be saved for emergency packets for mRSUs may be obtained using the related computations. Ultimately, from the perspective of the end-to-end delay and packet delivery ratio, the three-log distance and Friis models surpass the other models, while Nakagami, Rayleigh, and Rician have higher throughput.

## 5. Conclusions and Future Work

### 5.1. Conclusions

Utilizing energy collaboration between RSUs and the transfer of energy across RSUs and downstream vehicles, this research maximizes the network lifespan in vehicular ad hoc networks. Every RSU and vehicle in the vehicle-mounted ad hoc network must address the flow of data and energy usage at almost the same instance. In a VANET simulation, the propagation model utilized has a significant impact on the outcomes. It has an influence on which nodes are able to interact and the likelihood of receiving data correctly. As a result, it can affect the rate at which messages travel over the network, affecting the end-to-end delay in multi-hop scenarios. The overhead in terms of collisions and medium utilization is also influenced by the probability distribution of accurate reception. Since real-world implementation may differ from the simulation, caution must be exercised when mapping the model and parameters to the target environment. Because deterministic site-specific propagation modeling, such as in wireless systems site design, is not feasible in VANET simulations due to the variable nature of the environment and the mobility associated, the propagation environment is often treated stochastically. This study provides a performance analysis of VANET propagation models in terms of both vehicle count and packet size. For simulation, SUMO and NS-3.29 are utilized in this research work. The findings are presented and examined in depth. According to the outputs of the Friis, Nakagami, Rayleigh, Rician, and range propagation models, the AODV has good throughput, average delay, packet delivery ratio, hop count, and energy usage values. In practically every scenario, the mRSU is the best. The average delay and packet delivery ratio of the mRSU in the three-log distance and Friis models are demonstrated to be superior to those of others with a varied number of nodes (vehicles). When integrating the mobile RSU, the Nakagami, Rician, and Rayleigh models deliver superior outcomes in terms of throughput. The experimental computational findings suggest that collaborating for energy usage in VANET technology may significantly increase the network lifespan.

### 5.2. Future Work

Future research will focus on evaluating various routing protocols, such as OLSR and DSDV, with certain contemporary propagation models. A model will also be sought to improve the 802.11p MAC layer for the delay-tolerant transmission of emergency messages and congestion control techniques.

## Figures and Tables

**Table 1:** Energy consumption values for 300 KB message.

	I_1_	V_1_	P_1_ = VI	I_2_	V_2_	P_2_ = VI	P_1_ − P_2_	Time	Distance	Energy E = P × T	Energy per Day kJ/Day
1	480	4.01	1.92	362	4.00	1.44	0.47	2	60	0.9581	14.3721
2	445	4.02	1.78	314	4.01	1.26	0.52	20	130	10.581	158.724
3	467	4.00	1.86	332	4.00	1.32	0.54	40	200	21.642	324.636
4	483	3.99	1.92	336	4.01	1.34	0.58	60	400	34.860	522.909
5	468	4.01	1.87	308	4.01	1.23	0.64	80	600	51.428	771.432
6	470	4.00	1.88	330	4.02	1.32	0.55	100	650	55.401	831.015

**Table 2:** Summary of notations.

Notation	Description
${Th}_{avg}$	Average throughput
$P_{r\;}$	Packet received
$P_{s}$	Packet sent
$T_{l}$	Time of last received packet
$T_{f}$	Time of first transmit packet
*EED*	End-to-end delay
$T_{delay}$	Transmission delay
$P_{delay}$	Propagation delay
${Pro}_{delay}$	Processing delay
$Q_{delay}$	Queuing delay
*PDR*	Packet delivery ratio
$T_{Sim}$	Total simulation
kB	Packet size in kilobytes
kJ/day	Energy per day

**Table 3:** Simulation parameters.

Simulation Parameter	Value
Vehicles	49, 79, 109, 10, 30, 50
Mobile RSU	2, 4, 6
Dimensions	2000 × 2000 m
Initial Energy	100 J
Vehicles Speed	Random
RSU Speed	20 m/s
MAC Protocol	IEEE 802.11p
Packet Size (KB)	500, 1000, 1500, 2000, 2500, 100, 200, 300, 400, 500
Simulation Time in SUMO	360 s
Routing Protocol	AODV
Transmission Power	20 dBm
Transmission Range	200 m
Simulation Time in NS-3	150 s
Data Rate	1024 bps
Radio Range	DSRC
Frequency	5.9 GHz

## Data Availability

Not Applicable.
